# GATNNCDA: A Method Based on Graph Attention Network and Multi-Layer Neural Network for Predicting circRNA-Disease Associations

**DOI:** 10.3390/ijms22168505

**Published:** 2021-08-07

**Authors:** Cunmei Ji, Zhihao Liu, Yutian Wang, Jiancheng Ni, Chunhou Zheng

**Affiliations:** 1 School of Cyber Science and Engineering, Qufu Normal University, Qufu 273165, China; liuzhihao19971002@gmail.com (Z.L.); wytfuture@163.com (Y.W.); nijch@163.com (J.N.); 2 School of Artificial Intelligence, Anhui University, Hefei 230601, China

**Keywords:** circRNA–disease associations, graph attention network, multi-layer neural network

## Abstract

Circular RNAs (circRNAs) are a new class of endogenous non-coding RNAs with covalent closed loop structure. Researchers have revealed that circRNAs play an important role in human diseases. As experimental identification of interactions between circRNA and disease is time-consuming and expensive, effective computational methods are an urgent need for predicting potential circRNA–disease associations. In this study, we proposed a novel computational method named GATNNCDA, which combines Graph Attention Network (GAT) and multi-layer neural network (NN) to infer disease-related circRNAs. Specially, GATNNCDA first integrates disease semantic similarity, circRNA functional similarity and the respective Gaussian Interaction Profile (GIP) kernel similarities. The integrated similarities are used as initial node features, and then GAT is applied for further feature extraction in the heterogeneous circRNA–disease graph. Finally, the NN-based classifier is introduced for prediction. The results of fivefold cross validation demonstrated that GATNNCDA achieved an average AUC of 0.9613 and AUPR of 0.9433 on the CircR2Disease dataset, and outperformed other state-of-the-art methods. In addition, case studies on breast cancer and hepatocellular carcinoma showed that 20 and 18 of the top 20 candidates were respectively confirmed in the validation datasets or published literature. Therefore, GATNNCDA is an effective and reliable tool for discovering circRNA–disease associations.

## 1. Introduction

Circular RNAs (circRNAs) are a new class of endogenous non-coding RNA lacking a 5${}^{\prime }$ cap and a 3${}^{\prime }$ polyadenylated tail [1,2]. Since circRNAs were first discovered, in the 1970s, they have been considered as splicing errors [3,4]. In the past decade, with the development of high-throughput sequencing technology, a large number of circRNAs have been identified in mammalian cells [5,6]. Researchers have found that circRNAs are widely expressed in human tissues, and have stable structure and tissue-specificity. The mechanism of circRNA expression remains unknown, and how the biogenesis of circRNA affects its unique regulatory pattern remains limited [7]. Studies have revealed that many circRNAs perform their biological functions by acting as sponges of microRNA or RNA-binding proteins, by regulating protein function or by being translated themselves [8,9,10].

Cumulative evidence has indicated that many circRNAs are involved in human diseases, especially cancers [11]. For example, circHIPK3 has been found significantly up-regulated in colorectal cancer (CRC) tissues by sponging miR-7 to inhibit miR-7 activity [12]. Hsa_circ_0000190 was down-regulated in gastric cancer (GC) tissues and plasma from patients with GC. Compared with common biomarkers such as CEA and CA19-9, it has better sensitivity and specificity, and can be used as a novel biomarker for diagnosis of gastric cancer [13]. Researchers have identified that the expression of hsa_circ_0005075 is significantly different between hepatocellular carcinoma (HCC) and normal tissues [14]. The expression of Hsa_circ_0001649 was significantly different between HCC and normal liver tissues [15]. Moreover, circRNAs have also been related to other human diseases. CircANRIL is related to atherosclerotic disease by binding to pescadillo homolog 1 (PES1), which then impairs pre-rRAN processing and ribosomal biogenesis, results in the activation of p53, and thereby induces apoptosis and inhibits proliferation [14]. Recent studies have shown that the circRNA level in the brain is associated with Alzheimer’s disease (AD) [16]. Compared with the control group, Li et al. have found that 112 circRNAs were up-regulated and 51 circRNAs were down-regulated in AD patients [17], which also were enriched in AD-related pathways, and the clinical guidance of circ-AXL, circ-GPHN and circ-PCCA in disease management of AD patients was identified.

As researchers have realized that circRNAs are abundant in mammalian cells, evolutionarily conserved and stable, and could serve as better biomarkers [18], databases of rich circRNA information, such as circBase [19], circ2traits [20], CircFunBase [21] have been built for study. Furthermore, researchers have also manually curated evidence from published literature, established databases such as circRNADisease [19], CircR2Disease [22], Circ2Disease [23], and circAtlas [24]. While experimental verification is expensive and time-consuming, computational methods have gradually introduced inferring potential circRNA–disease associations. Lei et al. first proposed a path weighted method to predict disease-related circRNAs. They calculated disease semantic similarity, disease functional similarity and integrated with the Gaussian Interaction Profile (GIP) kernel similarities. Then, they constructed a heterogeneous network and adopted the depth-first search (DFS) to traverse nodes in the network and calculate the predictive score [25]. Yan et al. developed the DWNN-RLS method based on Regularized Least Squares of Kronecker product kernel for predicting circRNA–disease associations, and obtained AUC values of 0.8854, 0.9205 and 0.9701 in fivefold, 10-fold and leave-one-out cross validation, respectively [26]. Another graph-based method KATZHCDA achieved the best AUC values of 0.7936 and 0.8469 in fivefold CV and LOOCV, respectively [27]. Xiao et al. developed a weighted low-rank approximation optimization method with dual-manifold regulations to infer potential circRNA–disease associations [28].

Deep learning algorithms have also been introduced in this field. Deepthi et al. proposed an ensemble method named AE-RF, which extracted features via deep autoencoder, and then used random forest for prediction. As a result, this method achieved 0.9486 and 0.9552 in fivefold and 10 fold CV, respectively [29]. Li et al. used DeepWalk to extract node features in the circRNA–disease network, and used a network consistency projection algorithm for circRNA–disease interactions prediction [30]. Wang et al. designed GCNCDA using FastGCN to extract high-level features, and by applying Forest PA classifier for prediction [31]. As a result, it achieved an AUC value of 0.909 in fivefold CV based on circR2Disease dataset. Bian et al. developed GATCDA method based on graph attention network to obtain representation of circRNAs and diseases, calculated the probability score by dot production [32], and yielded an AUC value of 0.9011.

**Figure 1:**
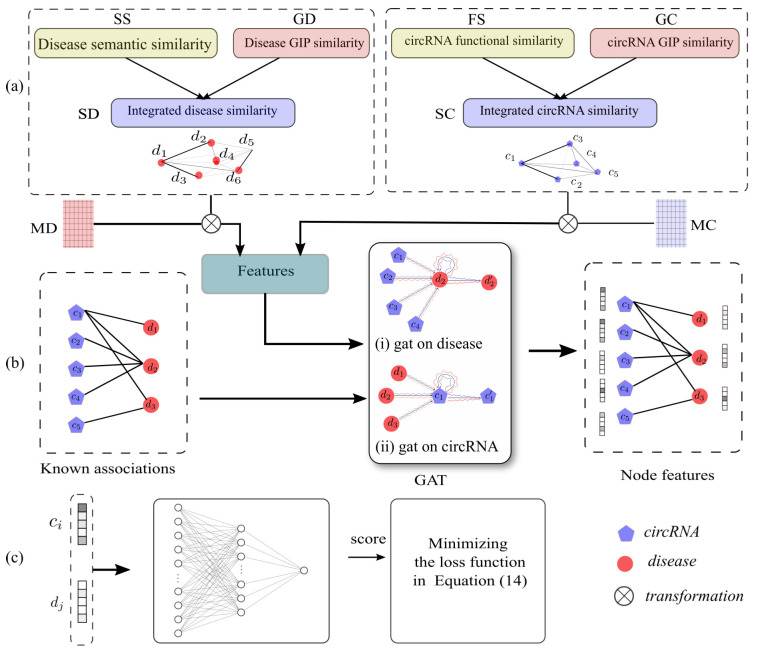
The framework of GATNNCDA. It consists of three steps: (**a**) similarity integration for circRNA and disease, (**b**) GAT-based feature extraction, and (**c**) NN-based classification.

In this study, we proposed a novel computational method named GATNNCDA to predict potential circRNA–disease associations, based on graph attention network and multi-layer neural network. To be specific, GATNNCDA first integrates circRNA functional similarity, disease semantic similarity and the GIP similarities. Secondly, GATNNCDA utilizes linear transformation to project the integrated similarity matrices into the same space, and applies a graph attention network to extract dense representations of nodes in the heterogeneous circRNA–disease graph. Furthermore, a multi-layer neural network is constructed to infer the associations between circRNAs and diseases. The framework of GATNNCDA is shown in Figure 1. In summary, our contributions are listed as follows:

- We proposed an end-to-end framework for inferring disease-related circRNAs, which can effectively and accurately infer the potential associations between circRNAs and diseases.
- We made use of GAT to extract low-dimensional dense representations of circRNAs and diseases, and these presentations had rich structural and semantic information of the heterogeneous circRNA–disease graph.
- We proposed a NN-based classifier, and applied a sampling strategy to construct balanced samples. In addition, we designed cross-entropy loss with L2 regularization to make the training process fast and robust.
- We demonstrated the predictive performance of our method by extensive experiments via fivefold cross validation and case studies, and achieved competitive results on CircR2Disease and circRNADisease datasets.

## 2. Results and Discussion

### 2.1. Experiments Settings

In our experiments, we conducted fivefold cross-validation (fivefold CV) to evaluate the prediction performance of GATNNCDA. In particular, we randomly split all samples into five groups, of which four of them were used for training and the other group for validation. Furthermore, we carried out several commonly used criteria in this field [33,34,35] to quantitatively analyze the performance of our method, such as accuracy, precision, recall and F1-score. Moreover, we also plotted the receiver operating characteristic curve (ROC) and precision-recall (PR) curve, and calculated the area under the ROC curve (AUC) and the area under the PR curve (AUPR).

The implementation of our method was based on Python machine learning library PyTorch v1.6.0 [36]. Graph attention network was developed by using PyTorch Geometric deep learning library [37]. We carried out our experiments on the Ubuntu 16.04, with two Tesla V100 GPUs. The default settings for GAT are 2 GAT layers and 4 heads. While the dimension size is set to 32, the classifier is 2-layer fully-connected layers. In addition, We used Adam optimizer [38] to update parameters of GATNNCDA iteratively.

### 2.2. Performance Analysis

**Figure 2:**
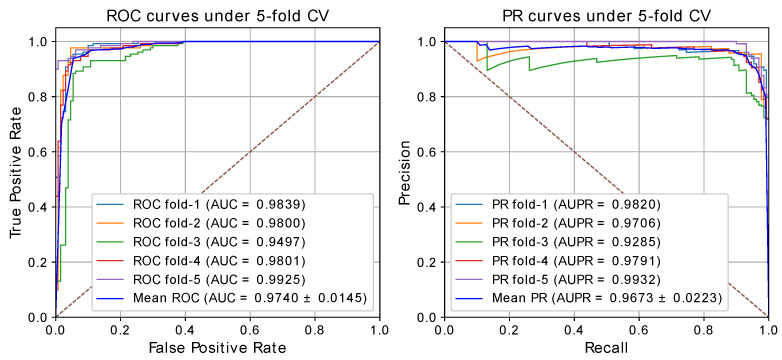
The framework of GATNNCDA.

To evaluate the performance of our method, we conducted the fivefold CV on CircR2Disease [22]. Here, $N_{c}=585$ and $N_{d}=88$ denote the number of circRNAs and diseases. We performed fivefold CV 50 times on CircR2Disease, and the best performance is shown in Table 1, with average accuracy of 0.9315, precision of 0.9714, recall of 0.9615, F1-score of 0.9336, AUC of 0.9742 and AUPR of 0.9707. We also plotted the ROC and PR curves as shown in Figure 2. The average AUC and AUPR values of 50 times are 0.9619 and 0.9452, respectively.

We also performed the fivefold CV on another commonly used circRNA–disease association dataset, cicRNADisease [19]. In circRNADisease, the number of circRNAs $N_{c}=313$, and the number of disease $N_{d}=44$. We can construct a circRNA–disease graph, calculate the similarities and train and validate GATNNCDA by similar criteria. The results are shown in Table 2. It can be seen that GATNNCDA obtained an average accuracy of 0.9638, precision of 0.9852, recall of 0.9910, F1-score of 0.9649, AUC of 0.9882 and AUPR of 0.9848. Therefore, the results on CircR2Disease and cicRNADisease showed that GATNNCDA performed well and can promote the prediction performance of potential disease-related circRNAs.

### 2.3. Comparison with Other Methods

As some methods have been proposed for inferring circRNA–disease association, we compared the performance of GATNNCDA with other state-of-the-art methods by fivefold CV. Some methods used different evaluation criteria or datasets. To compare fairly, we chose nine methods and mainly used CircR2Disease dataset and AUC as the criteria, including DWNN-RLS [26], PWCDA [25], KATZHCDA [27], NCPCDA [39], AE-RF [29], Wang’s method, [40], iCircDA-MF [41], GCNCDA [33] and GATCDA [29]. We performed the experiment 50 times, and selected the best performance and the average performance for comparison, denoted as GATNNCDA-best and GATNNCDA-average. The results are shown in Table 3. It can be seen that GATNNCDA is superior to the other nine methods. It is worth noting that the latter two methods are graph neural network based. We found that the data used in GCNCDA and GATCDA are not exactly the same as for us. GCNCDA uses all known circRNA–disease associations in the CircR2Disease dataset, while GATCDA integrates the data with other datasets. However, the AUC value of our method outperforms these methods by a large margin, which demonstrates that GCTNNCDA can effectively and accurately predict underlying disease-related circRNAs.

### 2.4. Ablation Study

In this section, we quantitatively evaluated the effect of different components, such as similarity integration, GAT-based feature extraction, and multi-layer NN-based classification, we performed the ablation study by using fivefold CV based on the CircR2Disease dataset. Specially, we defined the variants of GATNNCDA as follows:

- GATNNCDA w/o features: It uses randomly initialized SD and SC as initial node features, instead of integrated similarities.
- GATNNCDA w/o GAT: It removes the GAT from GATNNCDA, and uses the integrated similarities as features and a two-layer NN as a predictor.
- GATNNCDA w/o NN: It uses dot production to calculate the prediction score, instead of a two-layer NN as a predictor.

**Figure 3:**
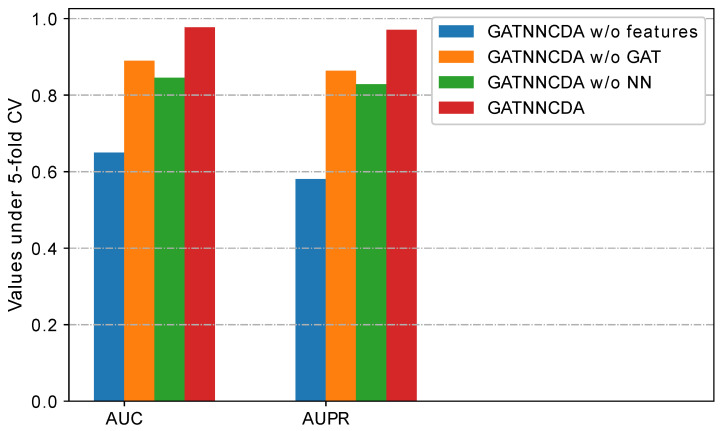
The comparison results of GATNNCDA and its variants based on CircR2Disease dataset.

The results are shown in Figure 3. GATNNCDA w/o features has the lowest values of AUC and AUPR, indicating that the integration similarities as initial node features can greatly improve the performance. GATNNCDA w/o GAT and GATNNCDA w/o NN have about 10% performance degradation. Therefore, our proposed method, GATNNCDA, combines the advantages of these components to obtain the best performance.

### 2.5. Effect of the Parameters

**Figure 4:**
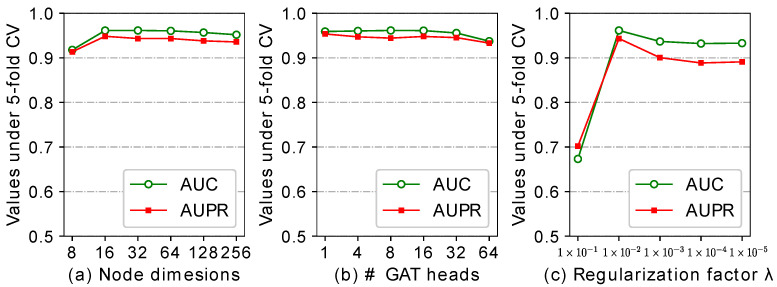
The comparison results of different parameter values based on CircR2Disease dataset.

GATNNCDA has several hyper-parameters that also affect the predictive performance. In this section, we performed the experiments to evaluate the effect of the parameters, such as the dimension size of nodes, number of heads in GAT, and the regularization factor, based on CircR2Disease dataset. Figure 4 shows the results of AUC and AUPR under different parameter values.

Recall dimension size of nodes not only affect the similarity parameter matrices MC, MD, but also impact the input features in the GAT and the NN-based classifier. In our experiment, we chose the values of {8, 16, 32, 64, 128, 256} to test the influence of dimension size. As shown in Figure 4a, we can see that GATNNCDA achieves the lowest AUC and AUPR when the dimension is set to 8, and obtains the best performance at 32. As the dimension increases beyond 32, the performance degrades slightly. The result demonstrate that too small dimensions could lead to under expression of diseases and circRNAs, while too large dimensions may lead to high noise. Therefore, we set 32 as our default dimension.

As reported in a previous study, the deeper GNN can degrade the performance [43]. We set 2 as the default number of the GAT layer. Then, we conducted an experiment on the different number of heads of GAT. Figure 4b shows that GATNNCDA achieves the best AUC at four GAT heads, and the best AUPR at one GAT head. Considering most methods use AUC as a criteria in performance comparison, we finally choose four as the default number of heads of GAT. In addition, we also designed the experiment to evaluate the regularization factor λ. As shown in Figure 4c, GATNNCDA acquires the best AUC and AUPR at $\lambda =1\times 10^{-2}$.

### 2.6. Case Studies

To further evaluate the prediction ability of our proposed method, we performed two case studies in this section. We trained GATNNCDA on CircR2Disease dataset [22], and then verified the candidates on circRNADisease [19] and circAtlas v2.0 [24] datasets. The first case study was conducted on breast cancer, which is one of the most common cancers in women. In particular, we constructed the positive samples with all known associations between circRNAs and diseases in the CircR2Disease. Meanwhile, we randomly chose the same number of negative samples from the unknown associations. Based on these training samples, we built the GATNNCDA and calculated the scores between breast cancer and each circRNA. Finally, we selected the top 20 related circRNAs for analysis. As shown in Table 4, 18 of the top 20 are confirmed by the validation datasets. The other two candidates have been verified in the recently published literature.

The second case study is performed on hepatocellular carcinoma. It is the most common form of liver cancer, with a higher incidence in patients with long-term liver diseases [44]. We utilized GATNNCDA to calculate the correlation score with circRNAs and then sorted by descending order. The top 20 hepatocellular carcinoma related cirRNAs are listed in Table 5. We can see that 10 of the top 20 are verified by the validation datasets, and the other eight candidates have been conformed in relevant literature, e.g., hsa_circ_0000520 is one of the three circRNAs that showed significantly different expression levels in HCC tissues [14]. Therefore, the unknown associations with high scores are likely to be correlated.

## 3. Materials and Methods

### 3.1. Known circRNA-Disease Associations

The experimentally verified circRNA–disease association dataset used in this paper is CircR2Disease [22]. We directly downloaded the dataset from the website (http://bioinfo.snnu.edu.cn/CircR2Disease, retrieved 7 June 2021). It contains 739 experimentally validated associations collected from some published studies, and includes 661 circRNAs and 100 diseases. After preprocessing, we obtained 585 circRNAs and 88 diseases. We defined the adjacent matrix $Y\in R^{N_{c}\times N_{d}}$ to denote the known circRNA–disease associations. The element $Y(c_{i},d_{j})$ is 1 if the association between circRNA $c_{i}$ and disease $d_{j}$ has been verified in CircR2Disease. Otherwise, $Y(c_{i},d_{j})$ is 0. $N_{c}=585$ and $N_{d}=88$ are the number of circRNAs and diseases.

### 3.2. Disease Semantic Similarity

We used the Disease Ontology dataset (DO) to calculate the similarity score between disease–disease pairs, which can be download from https://disease-ontology.org (retrieved 7 June 2021). Every disease has a term structure, including a unique ID, name, and the is-a relation with its parents. Given a disease *d*, we can build a Directed Acyclic Graph (DAG) represented as $DAG_{d}=\left(T_{d},E_{d}\right)$. $T_{d}$ and $E_{d}$ denote the nodes and edges in the $DAG_{d}$. Based on the assumption that the more shared the nodes in the DAGs between two diseases are, the more similar they are, we can calculate the semantic similarity between disease $d_{i}$ and $d_{j}$ using DOSE package, and denote matrix $SS\in R^{N_{d}\times N_{d}}$ as the semantic similarities between diseases.

### 3.3. circRNA Functional Similarity

As proposed in the previous work for computing functional similarity between miRNAs [45], we assumed that the more similar the diseases connected to two circRNAs, the more similar their functions will be [45]. In particular, we denoted circRNA functional similarity between circRNA $c_{i}$ and $c_{j}$ as $CS(c_{i},c_{j})$. Let $D_{i}$ and $D_{j}$ represent the related disease groups that were calculated from the known circRNA–disease associations. Then, we defined the functional similarity between circRNA $c_{i}$ and $c_{j}$ as following:

(1) $$FS\left(c_{i},c_{j}\right)=\frac{\sum_{d_{k}\in D_{j}}S\left(d_{k},D_{i}\right)+\sum_{d_{l}\in D_{i}}SS\left(d_{l},D_{j}\right)}{|D_{i}\left|+\right|D_{j}|}$$

where $S\left(d,D\right)=\max_{d_{i}\in D}\left(SS\left(d,d_{i}\right)\right)$ is the disease similarity between disease *d* and group *D*. $|D_{i}|$ and $|D_{j}|$ are the number of diseases in the group $D_{i}$ and $D_{j}$.

### 3.4. Gaussian Interaction Profile Kernel Similarity for Disease

Based on the assumption that similar circRNAs are more likely connected to similar diseases [46], we denoted *i*-row of *Y* and *j*-column of *Y* as the representations of circRNA $c_{i}$ and disease $d_{j}$, and then calculated the we Gaussian interaction profile (GIP) kernel similarities between two circRNAs or diseases as follows:

(2) $$\begin{array}{cc} GC(c_{i},c_{j}) & =\exp(-\gamma_{c}∥Y_{i\cdot }-Y_{j\cdot }{∥}^{2}) \end{array}$$

(3) $$\begin{array}{cc} GD(d_{i},d_{j}) & =\exp(-\gamma_{d}∥Y_{\cdot i}-Y_{\cdot j}{∥}^{2}) \end{array}$$

where $\gamma_{d}$ and $\gamma_{c}$ are the kernel bandwidth control parameters, and are defined by the following equations:

(4) $$\begin{array}{cc} \gamma_{c} & =\frac{1}{\frac{1}{N_{c}}\sum_{i=1}^{N_{c}}{∥Y_{i\cdot }∥}^{2}} \end{array}$$

(5) $$\begin{array}{cc} \gamma_{d} & =\frac{1}{\frac{1}{N_{d}}\sum_{j=1}^{N_{d}}{∥Y_{\cdot j}∥}^{2}} \end{array}$$

### 3.5. Integrated Similarities for circRNA and Disease

We observed that the similarity matrices SS and FS are very sparse. Therefore, we integrated GIP similarities to improve the expression of disease similarity and circRNA similarity. The formulas are as follows:

(6) $$SC\left(c_{i},c_{j}\right)=\left\{\begin{array}{cc} FS(c_{i},c_{j})\; & \mathrm{if}FS(c_{i},c_{j})\neq 0\\ GC(c_{i},c_{j})\; & \mathrm{otherwise} \end{array}\right.$$

(7) $$SD\left(d_{i},d_{j}\right)=\left\{\begin{array}{cc} SS(d_{i},d_{j})\; & \mathrm{if}SS(d_{i},d_{j})\neq 0\\ GD(d_{i},d_{j})\; & \mathrm{otherwise} \end{array}\right.$$

where $SC\in R^{N_{c}\times N_{c}}$ and $SD\in R^{N_{d}\times N_{d}}$ are integrated similarities.

### 3.6. Feature Extraction Based on Graph Attention Network

Graph attention network (GAT) is a powerful graph-based method whose node can aggregate its neighbor’s information by an attention mechanism [47]. In this section, we used GAT in the circRNA–disease graph to learn the rich representations of circRNAs and diseases. We first constructed the circRNA–disease graph based on adjacency matrix *Y*, and defined it as G=(V,E). $V=\{v_{1},v_{2},\cdots ,v_{N_{c}+N_{d}}\}$ are vertices, *E* represents the edges between circRNA and disease. In particular, edges in the circRNA–disease graph are un-directional, so *G* can be considered as a bidirectional graph.

As the integrated similarities SC and SD are in different dimension size, we introduced two parameter matrices $MC\in R^{N_{c}\times F}$ and $MD\in R^{N_{d}\times F}$ to transform SC and SD to the same size, and defined the initial node features in graph *G* as follows:

(8) X=concat(SC×MC,SD×MD)

where *F* is the dimension size, and concat denotes matrix concatenation. We denoted the input of *l*-layer of GAT as $H^{\left(l\right)}=\left\{h_{1}^{\left(l\right)},h_{2}^{\left(l\right)},\cdots ,h_{N}^{\left(l\right)}\right\},h_{i}^{\left(l\right)}\in R^{F^{\left(l\right)}}$, and we set $H^{\left(0\right)}=X$ as the initial input to GAT. In the circRNA–disease graph, some vertices have no connections with others. To keep the dimensions of GAT output the same as the dimensions of input node features, we set $F^{\left(l\right)}=F$. Then, we defined the coefficient between node $v_{i}$ and the neighborhood $v_{j}$ by the following formula:

(9) $$e_{ij}^{\left(l\right)}=a\left(W^{\left(l\right)}h_{i}^{\left(l\right)},W^{\left(l\right)}h_{j}^{\left(l\right)}\right)$$

where $W^{\left(l\right)}$ is the *l*-layer shared parameter, and *a* represents a single-layer neural network with LeakyReLU as the activation function. Similarly, we calculated the coefficients over the neighbor $N_{i}$, and normalized the score of node $v_{j}$ as follows:

(10) $$\alpha_{ij}^{\left(l\right)}=softmax_{j}^{\left(l\right)}\left(e_{ij}^{\left(l\right)}\right)=\frac{\exp(e_{i,j}^{\left(l\right)})}{\sum_{k\in N_{i}\exp\left(e_{ik}^{\left(l\right)}\right)}}$$

For node $v_{i}$, the output of *l*-layer over multi-head attention mechanisms can be defined as follows:

(11) $$h_{i}^{\left(l+1\right)}=\sigma \left(\overset{K}{\underset{k=1}{\|}}\sum_{j\in N_{i}}\alpha_{ij}^{\left(l,k\right)}W^{\left(l,k\right)}h_{j}^{\left(l\right)}\right)$$

where σ is a nonlinear activation function. *K* is the number of independent attention heads. ‖ denotes concatenation of *K* heads except averaging in the last GAT layer. As the *L*-layer GAT calculation, we obtained the final node features, and defined as $H^{\left(L+1\right)}=\left\{c_{1},c_{2},\cdots ,c_{N_{c}},d_{1},d_{2},\cdots ,d_{N_{d}}\right\}$.

### 3.7. circRNA-Disease Association Prediction

In this section, we constructed a NN classifier to predict the associations between circRNAs and diseases. The *k*-layer output of the NN classifier can be defined as follows:

(12) $$h^{\left(k+1\right)}=\sigma \left(W^{\left(k\right)}\times h^{\left(k\right)}+b^{\left(k\right)}\right)$$

where $h^{\left(0\right)}=concat\left(c,d\right)$ is the input to NN classifier, concatenated by the vectors of circRNA *c* and disease *d*. σ denotes LeakyReLU activation function. $W^{\left(k\right)}$ and $b^{\left(k\right)}$ are the parameters of weight and bias in the *k*-layer of NN classifier. In the last layer (*K*-layer) of the NN classifier, we can calculate the correlation score as follows:

(13) $$f\left(c,d\right)=h^{\left(K+1\right)}=\sigma \left(W^{\left(K\right)}\times h^{\left(K\right)}+b^{\left(K\right)}\right)$$

where σ is a sigmoid(·) activation function which ensure the score is between 0 and 1. In GATNNCDA, known pairs of circRNA and disease are taken as positive samples, and labeled as 1. However, there are no negative samples in the CircR2Disease; we randomly selected the same numbers of negative samples from the unknown associations, and marked them as 0. The training samples can be denoted as G. Finally, we can define our loss function by the following equation:

(14) $$\begin{array}{c} L=-\frac{1}{N}\sum_{(c,d)\in G}\left(y\log f\left(c,d\right)+\left(1-y\right)\log\left(1-f\left(c,d\right)\right)\right)+\lambda {∥\Theta ∥}^{2} \end{array}$$

where *N* is the number of training samples. λ denotes the control factor to the regularization, and Θ is the parameters of our model.

## 4. Conclusions

Cumulative evidence has shown that circRNAs play an important role in progression of human diseases, and are suitable as promising disease biomarkers for prevention, diagnosis and treatment. As traditional biological identification is very costly and time-consuming, more and more computational methods have been introduced in this field. In this study, we proposed a novel computational method called GATNNCDA for predicting potential circRNA–disease associations. GATNNCDA achieved a better performance than other state-of-the-art methods by combining similarity integration, graph attention network and multi-layer neural network. In particular, we performed fivefold CV for evaluation, and obtained the best performance of AUC of 0.9742, AUPR of 0.9707. The average values of AUC and AUPR for under 50 experiments were 0.9613 and 0.9452. Furthermore, case studies on breast cancer and hepatocellular carcinoma have also demonstrated that GATNNCDA can be a useful tool for predicting potential disease-related circRNAs.

However, GATNNCDA still has some limitations. The initial node features may not be perfect. Recall that similarity integration as initial node representations would affect the final performance. Nonetheless, known interactions between circRNA–disease associations are insufficient. In addition, circRNA functional similarity and GIP similarity may be inaccurate. Therefore, more biological information such as circRNA–miRNA association or circRNA sequence will be used for further study to construct more accurate node features, especially for some unseen circRNAs. Furthermore, the NN-based classifier of GATNNCDA requires negative samples for training, which are rarely reported in the literature. Randomly sampling from the unknown associations in a CircR2Disease dataset would introduce bias. In the future, we will seek a better negative sampling strategy to promote the performance of GATNNCDA.

## Figures and Tables

**Table 1:** Results of fivefold CV based on CircR2Disease dataset of best performance.

Test Fold	Accuracy	Precision	Recall	F1-Score	AUC	AUPR
1	0.9346	0.9821	0.9692	0.9368	0.9839	0.9820
2	0.9346	0.9720	0.9769	0.9373	0.9800	0.9706
3	0.9077	0.9305	0.9308	0.9098	0.9497	0.9285
4	0.9308	0.9793	0.9692	0.9333	0.9801	0.9791
5	0.9500	0.9933	0.9615	0.9506	0.9925	0.9932
Average	0.9315	0.9714	0.9615	0.9336	0.9742	0.9707

**Table 2:** Results of fivefold CV based on cicRNADisease dataset of best performance.

Test Fold	Accuracy	Precision	Recall	F1-Score	AUC	AUPR
1	0.9478	0.9799	1.0000	0.9504	0.9826	0.9794
2	0.9627	0.9944	0.9701	0.9630	0.9938	0.9943
3	0.9776	0.9719	1.0000	0.9781	0.9831	0.9703
4	0.9776	0.9879	0.9851	0.9778	0.9895	0.9877
5	0.9531	0.9921	1.0000	0.9552	0.9922	0.9920
Average	0.9638	0.9852	0.9910	0.9649	0.9882	0.9848

**Table 3:** The fivefold CV AUC comparison with the other nine methods based on CircR2Disease dataset.

Models	AUC
DWNN-RLS [26]	0.8854
PWCDA [25]	0.8900
KATZHCDA [27]	0.7936
NCPCDA [39]	0.9201
AE-RF [29]	0.9486
Wang’s method [40]	0.8667
iCircDA-MF [41]	0.9178
GCNCDA [31]	0.9090
GATNNCDA [42]	0.9011
GATNNCDA-best	0.9742
GATNNCDA-average	0.9613

**Table 4:** Top 20 predicted circRNAs related to Breast cancer based on circR2Disease dataset.

Rank	circRNA	Evidence	Rank	circRNA	Evidence
1	hsa_circ_0007534	*II*	11	hsa_circ_0068033	*I*; *II*
2	hsa_circ_0011946	*II*	12	circamotl1hsa_circ_0004214	*I*; *II*
3	hsa_circ_0093859	*II*	13	hsa_circ_0006528	*I*; *II*
4	circrna-000911	*II*	14	hsa_circ_0002874	*I*; *II*>
5	circrna-001283	PMID:29431182	15	hsa_circ_0001667	*I*; *II*
6	circrna-001175	*II*	16	hsa_circ_0085495	*I*; *II*
7	circrna-100438	PMID:29431182	17	hsa_circ_0086241	*I*; *II*
8	hsa_circ_0001982	*I*; *II*	18	hsa_circ_0092276	*I*; *II*
9	hsa_circ_0001785	*I*	19	hsa_circ_0003838	*I*; *II*
10	hsa_circ_0108942	*I*; *II*	20	circvrk1	*I*; *II*

*I*, *II* denote circRNADisease, circAtlas v2.0.

**Table 5:** Top 20 predicted circRNAs related to hepatocellular carcinoma based on circR2Disease dataset.

Rank	circRNA	Evidence
1	circc3p1	*II*
2	hsa_circ_0067531	*II*
3	circarsp91hsa_circ_0085154	*II*
4	circmto1hsa_circrna_0007874hsa_circrna_104135	*II*
5	hsa_circ_0005986	*I*; *II*
6	hsa_circrna_100338circsnx27	PMID:28710406
7	hsa_circrna_104075	*I*; *II*
8	hsa_circrna_102049	PMID:28710406
9	circrna_000839	*II*
10	circzkscan1hsa_circ_0001727	*I*; *II*
11	hsa_circ_0004018	*I*; *II*
12	hsa_circ_0005075	*II*
13	hsa_circrna_100571	PMID: 29609527
14	hsa_circrna_400031	PMID:29609527
15	hsa_circrna_102032	PMID: 29609527
16	hsa_circrna_103096	PMID:29609527
17	hsa_circrna_102347	PMID:29609527
18	hsa_circrna_000167hsa_circ_0000518	unknown
19	hsa_circ_0000520	PMID:27258521
20	hsa_circ_0000172	unknown

*I*, *II* denote circRNADisease, circAtlas v2.0.

## Data Availability

All data are present within the manuscript or available by request to corresponding author, Cunmei Ji (cunmeiji@126.com).
